# Acute Respiratory Distress Syndrome Caused by Influenza B Virus Infection in a Patient with Diffuse Large B-Cell Lymphoma

**DOI:** 10.1155/2011/647528

**Published:** 2011-10-29

**Authors:** Silvio A. Ñamendys-Silva, María O. González-Herrera, Julia Texcocano-Becerra, Angel Herrera-Gómez

**Affiliations:** ^1^Department of Critical Care Medicine, Instituto Nacional de Cancerología, 14080 Mexico City, Mexico; ^2^Department of Critical Care Medicine, Instituto Nacional de Ciencias Médicas y Nutrición Salvador Zubirán, 14000 Mexico City, Mexico; ^3^Department of Surgical Oncology, Instituto Nacional de Cancerología, 14080 Mexico City, Mexico

## Abstract

Influenza B virus infections are less common than infections caused by influenza A virus in critically ill patients, but similar mortality rates have been observed for both influenza types. Pneumonia caused by influenza B virus is uncommon and has been reported in pediatric patients and previously healthy adults. Critically ill patients with pneumonia caused by influenza virus may develop acute respiratory distress syndrome. We describe the clinical course of a critically ill patient with diffuse large B-cell lymphoma nongerminal center B-cell phenotype who developed acute respiratory distress syndrome caused by influenza B virus infection. This paper emphasizes the need to suspect influenza B virus infection in critically ill immunocompromised patients with progressive deterioration of cardiopulmonary function despite treatment with antibiotics. Early initiation of neuraminidase inhibitor and the implementation of guidelines for management of severe sepsis and septic shock should be considered.

## 1. Introduction

Influenza B virus infections are less common than infections caused by influenza A virus in critically ill patients, but similar mortality rates have been observed for both influenza types [1]. The pulmonary complications related with influenza include primary viral pneumonia, secondary bacterial pneumonia, and pneumonia attributable to unusual pathogens [2]. Pneumonia caused by influenza B virus is uncommon and has been reported in pediatric patients [3] and previously healthy adults [4]. From 22 May 2011 to 4 June 2011, National Influenza Centres (NICs) from 65 countries reported as positive for influenza viruses 483 specimens of which 233 (48.2%) were typed as influenza B [5]. At the beginning of 2011, influenza B has taken over influenza A H1N1 virus as the most dominant strain in circulation in some countries like United Kingdom [6]. We describe the clinical course of a critically ill patient with diffuse large B-cell lymphoma nongerminal center B-cell phenotype who developed acute respiratory distress syndrome (ARDS) caused by influenza B virus infection.

## 2. Case Report

A 69-year-old woman with a medical history of hypothyroidism was treated with levothyroxine. She was diagnosed with diffuse large B-cell lymphoma nongerminal center B-cell phenotype with nasopharyngeal involvement stage IVE (international prognostic index >3) on September 22, 2010. The immunohistochemical study from cervical lymph node was positive for CD3, CD20, B-cell lymphoma (BCL) 2, BCL-6, and multiple myeloma oncogene 1; tumor cells were negative for CD10. She was treated with 5 cycles of rituximab, cyclophosphamide, doxorubicin, vincristine, and prednisone (R-CHOP). In November 2010, the patient received the last cycle of R-CHOP. Over the last two months, her absolute lymphocyte count was <1.0 × 10^9^/L. She had not received influenza vaccine. 

**Figure 1:**
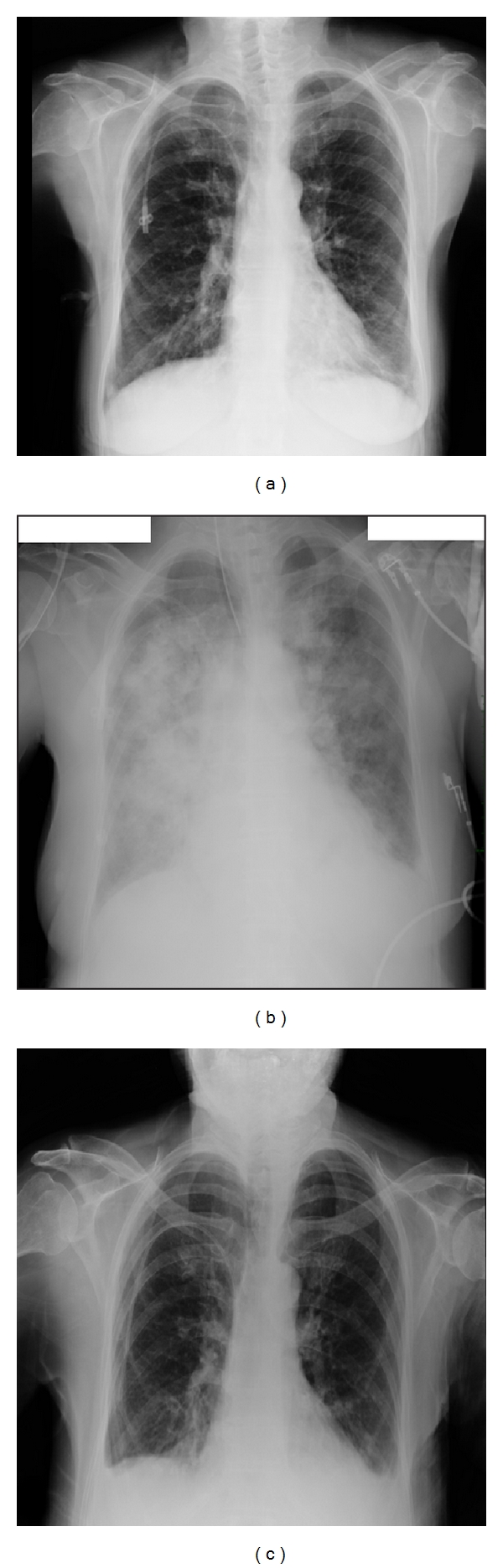
Chest radiographs of a patient with acute respiratory distress syndrome primary caused by influenza B virus infection. (a) On admission, no pulmonary infiltrates were found. (b) Chest radiograph shows bilateral diffuse alveolar opacities. (c) Chest radiograph a day prior to discharge shows complete resolution of the bilateral diffuse alveolar opacities.

On December 28, 2010, she was admitted to The National Cancer Institute located in Mexico City. On admission, she related a history of mild nonproductive cough and fever (38.8°C) for 2 days. On admission, no pulmonary infiltrates were found in the patient on chest radiograph (Figure 1(a)), her leucocytes count was 0.8 × 10^9^/L, and the SpO2 was 90% at room air. She was treated with ceftriaxone and clarithromycin. For the next 2 days, the patient persisted with fever and developed acute respiratory failure, and due to her critical condition, she was admitted to Intensive Care Unit (ICU); chest radiograph showed bilateral diffuse alveolar opacities consistent with pneumonia (Figure 1(b)), and due to further respiratory deterioration (PaO_2_/FIO_2_  ratio was 91 mm Hg), the patient was intubated and invasive mechanical ventilation was started. She required 8 cm H_2_O of positive end-expiratory pressure. The acute physiology and chronic health evaluation (APACHE) II and sequential organ failure assessment scores on admission to ICU were 16 and 11 points, respectively. 

She was treated for septic shock and primary acute respiratory distress syndrome (ARDS). Real-time polymerase chain reaction (RT-PCR) of nasopharyngeal swab, expectoration, and blood cultures were taken, and the antibiotic therapy was empirically changed to meropenem every 8 hours for ten days; the clarithromycin was stopped by an increase in liver functioning tests. Due to clinical suspicion of influenza virus and bacterial coinfection, oseltamivir 75 mg every 12 hours was added on day 2 after admission and continued for 10 days. Expectoration and blood cultures were negative, but the RT-PCR was positive for influenza B virus. She received treatment with vasoactive drugs and stress dose corticosteroids for 5 days. The patient progressively improved, and she was successfully weaned from mechanical ventilation after 6 days. The lengths of ICU and hospital stays were 10 and 17 days, respectively. Chest radiograph a day prior to discharge showed complete resolution of the bilateral diffuse alveolar opacities (Figure 1(c)). After further recovery, the patient was discharged home. 

## 3. Discussion

This patient with diffuse large B-cell lymphoma nongerminal center B-cell phenotype presented ARDS primary caused by influenza B virus infection and septic shock. 

Patients with pneumonia caused by influenza virus may develop acute respiratory failure and ARDS. During the 2009 H1N1 influenza pandemic, influenza (H1N1) A virus infection was an important cause of acute lung injury or ARDS [7]. 

The pathophysiologic mechanisms for the development of ARDS are still not fully understood [8]. A mouse model developed by Smith et al. [9] characterized the pathology and immunology of influenza-infected mice with severe secondary bacterial pneumonia and sepsis. They found elevated levels of both pro- and anti-inflammatory molecules. These elevated levels may induce tissue damage and interfere with the ability of the host to clear the inciting organisms [9]. During the acute phase of ARDS, there is an intense inflammatory process in the lung with sequential activation of cytokines, chemokines, and secretion of proteases, as well as concomitant collagen synthesis. On the other hand, the early phase of ARDS is characterized by increased permeability edema, interpreted as accumulation of protein-rich edema fluid into the air spaces [10]. 

Recently, Shieh et al. [11] described pathological studies on autopsy samples from 100 patients with fatal 2009 H1N1 virus infection that occurred during 2009 in the United States. The most frequent histopathological findings in lung tissues were edema (63%), hyaline membranes (59%), hemorrhage (58%), and inflammation (48%). In their paper [11], the 74% of case patients did not have confirmatory test results of bacterial coinfection. The clinical presentation of the critically ill patients with influenza B infection is characterized by acute respiratory failure, refractory hypoxemia, and bilateral alveolar infiltrates on chest radiograph [3, 4]. Approximately 70% of critically ill patients with influenza (H1N1) A virus infection required mechanical ventilation [2], and they had high mortality rate [12–14]. Influenza B virus has been identified (14%) in critically ill patients admitted to ICU [1, 15]. Furthermore, high prevalence of ARDS (23%) and mortality rate greater than 50% [1] in patients with influenza have been described.

The patients who are immunocompromised may have severe influenza infection. In a retrospective study [16] that included 100 immunocompromised patients with influenza, only 20% had influenza B virus infection. In this study [16], 60.4% of patients with pneumonia were admitted to ICU, of these patients, 34.3% required mechanical ventilation and 45.4% died.

In clinical practice, the mechanical ventilation strategies for patients with ARDS caused by influenza virus infection should be similar to those used in patients with ARDS due to other causes. The current evidence suggests that lung protective ventilation should be implemented in ventilator management of patients with ARDS because this strategy has demonstrated a significant mortality benefit [17]. Our patient received ventilatory management with this strategy which includes a target tidal volume of 6 mL/kg (predicted body weight) and the initial upper limit goal for plateau pressures ≤30 cm H_2_O. 

In this paper, the patient showed clinical deterioration despite treatment with antibiotics and oseltamivir was started. The ARDS and fever could have been caused by other infections that were not detected, since most laboratory tests were carried out after antibiotic treatment was initiated. The oral neuraminidase inhibitor oseltamivir has been studied at doses of 75 mg or 150 mg twice daily for five days in immunocompetent subjects with seasonal influenza [18]. Both dose levels of oseltamivir resulted in statistically significant reductions in the duration and severity of illness among those infected with influenza virus [18]. Prolonged influenza virus respiratory tract infection (A H3N2, A H1N1, and B) has been observed in immunocompromised patients with lymphopenia and is associated with development of influenza lower respiratory tract infection [19, 20]. The recommended dose is 75 mg twice daily for five days; however, longer duration and double dose of oseltamivir could be considered in immunosuppressed patients [21, 22]. Recent data published by Ariano et al. [23] suggest that a higher dose of oseltamivir is unlikely to be necessary in such cases. Oseltamivir was well absorbed enterically in critically ill patients with suspected or confirmed pandemic (H1N1) influenza who required admission to the ICU. The dosage of oseltamivir 75 mg twice daily achieved trough concentrations for the carboxylate metabolite that are well above any pharmacodynamic probability threshold for maximum inhibition of virus [23]. Early initiation (within 48 to 96 hours from onset) of oseltamivir for patients with influenza infection requiring hospitalization has been associated with shorter length of hospital stay [24] and improved survival [25]. 

Schnell et al. [16] recently reported that in immunocompromised patients with influenza, hematological malignancies, and influenza A infection independently predicted pneumonia. Furthermore, the development of ARDS and the history of immunosuppression have been described as independent risk factors for hospital mortality in critically ill patients with confirmed influenza virus infection [1]. Critically ill patients with complicated influenza pneumonia with an APACHE II score >20 and a PaO_2_/FiO_2_ ratio <150 have increased risk of death [26]. 

Our ICU routinely implements the guidelines for management of severe sepsis and septic shock [27, 28], because they have been associated with a significant reduction in hospital mortality in patients with severe sepsis and septic shock from 37% to 30.8% (*P* = 0.001) [29]. 

## 4. Conclusion

This paper emphasizes the need to suspect influenza B virus infection in critically ill immunocompromised patients with progressive deterioration of cardiopulmonary function despite treatment with antibiotics. Early initiation of neuraminidase inhibitor and the implementation of guidelines for management of severe sepsis and septic shock should be considered.
